# Alginate-like exopolysaccharides extracted from different waste sludges exhibit varying physicochemical and material properties

**DOI:** 10.3389/fmicb.2024.1493782

**Published:** 2024-11-05

**Authors:** Zahid Ur Rehman, Masoud Ghaani, Ahmed Y. A. Mohamed, John Gallagher, Pascal E. Saikaly, Muhammad Ali

**Affiliations:** ^1^Biological and Environmental Science and Engineering Division, Water Desalination and Reuse Research Center, King Abdullah University of Science and Technology, Thuwal, Saudi Arabia; ^2^Research and Analytical Services Department, Saudi. Aramco, Dhahran, Saudi Arabia; ^3^Department of Civil, Structural and Environmental Engineering, Trinity College Dublin, The University of Dublin College Green, Dublin, Ireland; ^4^TrinityHaus Trinity Research Centre, Trinity College Dublin, Dublin, Ireland

**Keywords:** aerobic granular sludge, alginate-like exopolysaccharides, activated sludge, membrane bioreactor, physicochemical characterization, wastewater

## Abstract

This study examined the influence of different wastewater treatment processes on the physicochemical properties of Alginate-Like Exopolymers (ALE) extracted from waste sludge. Sludge samples were collected from wastewater treatment plants (WWTPs) processing both combined industrial and domestic wastewater, as well as domestic wastewater alone. Among the processes studied, aerobic granular sludge (AGS) produced the highest ALE yield (352 ± 50 mg/g-VS_sludge_), significantly exceeding that from membrane bioreactor (170 ± 41 mg/g-VS_sludge_) and conventional activated sludge (<130 mg/g-VS_sludge_). AGS-derived ALE also had the highest uronic acid content (224 ± 14.5 mg/g-VS_sludge_), with mannuronic acids playing a critical role in enhancing hydrogel cohesion and stability. The results showed that the distinct microbial consortium in the AGS system, including the presence of *Pseudomonas alcaligenes*, was strongly associated with increased ALE production. This establishes a novel link between microbial community composition and ALE yield. These insights are crucial for optimizing resource recovery in AGS systems and underscore the potential of ALE for various industrial applications.

## 1 Introduction

Surplus waste sludge generated from wastewater treatment plants (WWTPs) poses significant management, transportation, and disposal challenges that can account for up to 50% of the overall cost of WW treatment (Lin et al., 2015). Given the increasing population and the rising pressure on finite resources, it is imperative to extract valuable resources present in waste sludge to promote a circular economy and sustainability. Among many resources, like phosphate, cellulose, and bioplastics, Alginate-like exopolymers (ALEs) have gained attention due to their unique properties, including strong gelling ability, biocompatibility, and biodegradability, which make them highly valuable for industrial applications (Ali et al., 2022). Such resource recovery from waste sludge can achieve a 50% reduction in operational expenditures at a WWTP, realize a 35% decrease in sludge production, and reduce carbon emissions (Ferreira et al., 2021). For instance, by 2030, the Netherlands anticipates recovering 85 Kilotons of ALE from waste sludge *per annum*, translating to a value of €170 million (van Leeuwen et al., 2018).

ALEs are extracellular polysaccharides produced by various microorganisms, including bacteria and algae. Bacterial-derived ALEs are composed of a mixture of biopolymers, including polysaccharides, proteins, humic acids, and lipids, and are typically extracted from bacterial aggregates through alkaline treatment followed by acid precipitation (Felz et al., 2016; Schambeck et al., 2020a). Alginate, a naturally occurring anionic polymer commercially produced by brown seaweed and also by certain bacterial species is the primary component of ALE. Extensive utilization of alginate in various biomedical applications, such as wound healing, drug delivery, and tissue engineering, can be attributed to its biocompatibility, low toxicity, and relative affordability (Lee and Mooney, 2012). Furthermore, alginate serves as a gelling agent in fields like textile printing, cosmetics, and the paper industry. In alginate molecules, the uronic acids—mannuronic acid (M) and guluronic acids (G)—can be arranged as either homopolymeric (MM or GG) or heteropolymeric (MG) blocks (Larsen et al., 1965). The physical and chemical properties of alginates are largely determined by the M and G content and their distribution along the alginate molecule. For instance, an increased G content leads to higher gelling characteristics, whereas a rise in M content correlates with increased viscosity. An elastic gel can be achieved with a high M/G ratio, while a low M/G ratio results in brittle gels. In general, G-blocks stably cross-link with divalent ions to form strong gels, whereas MM-and MG-blocks impart flexibility to the chains (Andriamanantoanina and Rinaudo, 2010).

ALEs exhibit properties that make them applicable in diverse fields, including agriculture, horticulture, medical, and construction industries (Lin et al., 2015; van Leeuwen et al., 2018). Several factors were suggested to determine ALEs secretion in biological systems, including reactor operating conditions, WW composition, microbial community composition, and extraction protocol (Schambeck et al., 2020a; Ding et al., 2015; Tu et al., 2012; Yang et al., 2019). While significant progress has been made in understanding ALE recovery, only a limited number of studies have examined its extraction from various wastewater treatment processes. For example, research has shown that ALE can be recovered in high concentrations from aerobic granular sludge (AGS), with yields significantly surpassing those from flocculant sludge (Lin et al., 2013; Schambeck et al., 2020a; Sarvajith and Nancharaiah, 2023). Furthermore, ALE derived from AGS has been shown to exhibit higher content and more stable properties, although its characteristics remain comparable to those of ALE from flocculant sludge (Schambeck et al., 2020b). In terms of improving recovery techniques, one study demonstrated that using sodium percarbonate instead of sodium carbonate increased ALE extraction efficiency by 30%, with the resulting biopolymers offering potential for biomedical applications due to their galactose and glucose composition (Liu et al., 2023). Additionally, the simultaneous recovery of phosphorus and ALE from different types of AGS highlights the role of sludge composition in optimizing resource recovery. For instance, algal-bacterial AGS was found to produce nearly three times more ALE than bacterial AGS (Chen et al., 2022). Reviews have also emphasized the economic and environmental advantages of ALE recycling from municipal sludge, underscoring the need for sustainable, product-focused approaches (Cheng et al., 2024). Moreover, findings from polyhydroxyalkanoate recovery studies have contributed to the broader understanding of biopolymer recovery in wastewater systems, further supporting the valorization of sludge (Pei et al., 2022). From an economic perspective, comprehending the composition and properties of ALE extracted from waste sludge generated by different wastewater treatment processes is crucial. Therefore, it prompts questions as to whether ALE from different wastewater treatment processes exhibits similar or distinct physical and chemical compositions.

Hence, this research aims to investigate variations in the quantity and composition of ALEs extracted from waste sludge, depending on the wastewater treatment process employed. Furthermore, we explore the physicochemical and material attributes of these ALEs and their impact on hydrogel-forming capabilities. For this study, ALEs were obtained from waste sludge collected from two full-scale conventional activated sludge (CAS) plants—one treating combined industrial and domestic wastewater and the other only domestic wastewater—as well as from a full-scale membrane bioreactor (MBR) plant and a pilot-scale AGS plant treating domestic wastewater. Advanced analytical techniques were employed to investigate the chemical and physical properties of the ALEs. It is hypothesized that ALE extracted from AGS exhibits higher yield and superior hydrogel-forming capabilities compared to ALE from CAS and MBR, due to the distinct microbial community and operational conditions in AGS systems. These insights will help us better understand the effect of wastewater sources on ALE physico-chemical properties and identify potential applications of ALE.

## 2 Materials and methods

### 2.1 Sample collection

Sludge samples were collected from different types of wastewater treatment plants, selected to encompass a range of treatment technologies and influent compositions, providing a representative comparison of sludge characteristics. Samples were obtained from a CAS plant treating combined industrial and domestic wastewater, located in the south of Jeddah (GPS coordinates: 21°22′27.8″N 39°13′36.2″E). This plant was included due to its treatment of mixed industrial and domestic wastewater, which results in a more heterogeneous sludge composition. This facility utilizes thermal sludge drying. Therefore, we collected sludge samples before and after the thermal sludge drying process, labeled as AK Sludge (undried) and AK Dry (dried). Additionally, samples were collected from another CAS plant solely treating domestic wastewater, situated in the north of Jeddah (GPS coordinates: 21°39′01.7″N 39°12′03.2″E), selected to provide a comparison with sludge generated from purely domestic wastewater treatment. Similar to the previous plant, we obtained samples before and after thermal treatment, named AP Sludge (undried) and AP Dry (dried). Furthermore, sludge samples were acquired from a full-scale MBR facility at KAUST University (GPS coordinates: 22°17′54.7″N 39°07′08.6″E), treating domestic wastewater, referred to as MBR sludge. The inclusion of the MBR plant aimed to compare advanced treatment processes with conventional ones in terms of ALE recovery potential. Finally, samples were collected from a pilot-scale AGS system, referred to as AGS sludge, (located in KAUST research labs) treating similar wastewater generated by the KAUST community, to assess the performance of this emerging technology in producing ALE, given its distinct operational conditions and microbial community structure.

### 2.2 Extraction of alginate-like exopolymers, ionic hydrogel formation and stability test

The ALE extraction followed the protocol outlined by Schambeck et al. (2020a). Initially, 3 grams of wet sludge was centrifuged at 4,000 *g* for 20 min at 4°C, discarding the resulting supernatant. The sludge pellet was then moved to a baffled flask equipped with a magnetic stirrer, where anhydrous Na_2_CO_3_ was added at proportions of 3:50:0.25 [sludge mass (g), deionized water (mL), and Na_2_CO_3_ (g)]. The mixture was stirred for 35 min at 400 rpm and 80°C in a water bath. After stirring, the supernatant containing soluble Extracellular Polymeric Substances (EPS) was isolated by centrifugation twice: first at room temperature (23°C) and then at 4°C, both at 4,000 g, for 35 and 20 min, respectively. This supernatant underwent dialysis using tubing with a 3,500 Da molecular weight cut-off (MWCO) (Spectrum^™^ Spectra/Por^™^), against ultrapure water for 24 h. To extract acidic ALE, its pH was adjusted to 2.2 ± 0.05 using 1 M HCl, stirred at 100 rpm. After another centrifugation step at 4,000 g for 20 min at 4°C, a gel-like pellet was isolated. Neutralizing this to obtain sodium-form ALE involved gradually adding 0.5 M NaOH with continuous stirring until a pH of 8.5 ± 0.05. The ALE measurements were conducted on a minimum of three samples and normalized with the volatile suspended solid (VSS) content of the sample as delineated by Felz et al. (2016). The total solids (TS) and volatile solids (*VS*) of the ALE were measured using standard methods (American Public Health Association, 2017).

After the dialysis process, the ALE was transferred to a glass beaker and incubated at 60°C until its volume diminished to 1–2 mL, thereby concentrating the polymer. To adjust the pH of the concentrated extract, 0.1 M NaOH was gently added with continuous stirring until a pH of 8.5 was achieved. Following this, the extract was gradually dripped into a 2.5% (w/v) calcium chloride solution, as described by Ali et al. (2015). Notably, when extracts were introduced to the calcium chloride solution, they instantaneously transformed into spherical beads. The stability of the ionic hydrogel beads was assessed by initially incubating them in CaCl_2_ for 30 min. Following this, the beads were carefully retrieved with a spoon and exposed to four distinct treatment conditions: MilliQ water, EDTA, formaldehyde, and formamide. These conditions were specifically selected to mimic various environmental and industrial scenarios where hydrogels may be exposed to different chemical treatments. The first set of beads was incubated in MilliQ water for 3 h at 4°C, serving as a control to assess the stability of the beads in a neutral, aqueous environment, representative of pure water conditions often encountered in environmental or industrial settings. The second set of beads was incubated in a 2% (W/V) EDTA solution for an identical duration and temperature. EDTA, a known chelating agent, was chosen to test the impact of metal ion removal (e.g., calcium) from the hydrogel structure. This simulates conditions in wastewater treatment plants where metal ions may be chelated and removed, potentially weakening the hydrogel network. The third group underwent treatment in a solution consisting of 14.3 mL of MilliQ and 120 μL of 37% formaldehyde for 1 h at 4°C. This was followed by the introduction of 5.7 mL of 1 M NaOH, with a continued incubation for an additional 2 h at 4°C. Formaldehyde was included to evaluate the hydrogel’s resilience in the presence of chemical preservatives, commonly used in laboratory and industrial applications for biological material stabilization. Finally, the fourth set of beads was incubated in a solution consisting of 14.3 mL of MilliQ and 120 μL of 99.5% formamide for 1 h at 4°C, followed by the addition of 5.7 mL of 1 M NaOH and an additional 2 h of incubation at 4°C. Formamide was used to simulate environments containing organic solvents, which are frequently found in industrial effluents and could impact the hydrogel’s integrity in such chemical-rich settings.

### 2.3 Measurement of proteins, humic substances, and polysaccharides

The quantification of proteins, humic substances, and polysaccharides within ALE required the deployment of multiple analytical techniques. For protein quantification, the Bradford protein assay was used for measurement (Bradford, 1976). This assay operates on the principle that the Coomassie Brilliant Blue G-250 dye binds to protein molecules, resulting in a color change that can be quantified spectrophotometrically at a wavelength of 595 nm. Bovine serum albumin was used as a standard to generate a calibration curve in which protein concentrations were calculated based on the absorbance values. In the case of humic substances, a standard curve was developed using humic acid in concentrations varying from 0 to 100 mg/L.

For the standards, glucose (0 to 100 mg/L) and glucuronic acids (0 to 400 mg/L) were used. All measurements were conducted in triplicates. The concentration of neutral sugars, denoted by [Glc], was expressed in glucose equivalents, while the concentration of uronic acids, denoted by [Gla], was expressed in Gla equivalents. The concentration of polysaccharide in ALE was presented as mg equivalent of Glu or Gla per gram of volatile solids (VS).

Samples and standards were mixed with a solution of 0.5 M NaOH and 0.5 M Na_2_CO_3_ and incubated at 40°C for 24 h. After incubation, the absorbance was measured at a wavelength of 400 nm, and the concentrations of humic substances were calculated using the established standard curve. To analyze both neutral sugars and uronic acids in a single assay, a method adapted from Rondel et al. was employed (Rondel et al., 2013). In this method, a 2 g/L anthrone reagent was prepared in 98% sulfuric acid. Subsequently, 200 μL of this solution was added to 100 μL of the sample or standard in a 48-well microplate. The plates were then incubated at 60°C for 30 min, and absorbance was measured at 560 and 620 nm at room temperature. It was observed that uronic acids exhibited a higher absorbance at 560 nm, while neutral sugars demonstrated a higher absorbance at 620 nm.

### 2.4 Nuclear magnetic resonance (NMR) analysis of EPS

The chemical composition of the extracted ALE was determined using ^13^C NMR spectrometry as described recently (Ali et al., 2018; Rehman et al., 2019). Solid-state ^13^C NMR spectra of EPS were obtained using 900 MHz AVANCE III NMR spectrometer (Bruker BioSpin, Germany) using a triple-resonance 3.2 mm Bruker MAS probe. The temperature was maintained at 25°C. Bruker Topspin 3.5p17 software (Bruker BioSpin, Germany) was used for data collection and processing of spectra.

### 2.5 DNA extraction, library preparation and bioinformatic analysis

To extract DNA from a 500 μL sample, the FastDNA Spin kit for soil (MP Biomedicals, USA) was employed using manufacturer instructions. After the extraction, the concentration of DNA was determined utilizing the Qubit dsDNA HS/BR Assay kit supplied by Thermo Fisher Scientific, USA. To prepare the library, the archaea/bacteria 16S rRNA gene variable region 4 (abV4-C) was amplified using Polymerase Chain Reaction (PCR) with an Illumina custom protocol (Illumina, 2013). For each PCR reaction (25 μL), 10 ng of template was used, along with 12.5 μL of PCRBIO Ultra mix, and 400 nM of each forward and reverse primer. The program included an initial denaturation step (95°C for 2 min), followed by amplification for 30 cycles (95°C for 15 s, 55°C for 15 s, and 72°C for 50 s), and a final elongation step at 72°C for 5 min following the method delineated by Albertsen et al. (2015). Primers with tails, designed by Apprill et al. (2015), had the sequences [515FBF] GTGYCAGCMGCCGCGGTAA and [806RB] GGACTACNVGGGTWTCTAAT. Libraries were purified using CleanNGS SPRI beads (CleanNA, NL) with a bead-to-sample ratio of 4:5. DNA was eluted in 25 μL of nuclease-free water provided by Qiagen, Germany, and the DNA concentration was determined using the Qubit dsDNA HS Assay kit (Thermo Fisher Scientific, USA). The product purity and size were confirmed using gel electrophoresis with Tapestation 2200 and D1000/High sensitivity D1000 screentapes (Agilent, USA).

Sequencing libraries were prepared using a second PCR of the purified amplicon libraries. PCR reaction (25 μL) comprised 10 ng of amplicon library template, PCRBIO HiFi buffer (1×), PCRBIO HiFi polymerase (1 U/reaction) (PCRBiosystems, UK), and an adaptor mix (400 nM of each forward and reverse). The program included an initial denaturation at 95°C for 2 min, amplification for 8 cycles (95°C for 20 s, 55°C for 30 s, and 72°C for 60 s), and a final elongation at 72°C for 5 min. The resulting sequencing libraries were purified using CleanNGS SPRI beads as described earlier. The Concentration, size, and purity of sequencing libraries were evaluated using the Qubit and gel electrophoresis as mentioned above. Finally, the libraries were purified and quantified as described above, and pooled in equimolar concentrations before being diluted to 2 nM. Paired-end sequencing was carried out on a MiSeq platform (Illumina, USA) with a 2 × 300 bp configuration, utilizing the MiSeq Reagent kit v3 (Illumina, USA). To address low complexity issues, a PhiX control library was spiked in at over 10%.

Quality filtering of the raw sequencing reads was carried out with Trimmomatic v. 0.32 (Bolger et al., 2014), using the parameters SLIDINGWINDOW:5:3 and MINLEN:225. Subsequently, the forward and reverse reads were merged with FLASH v. 1.2.7 with the settings-m 10-M 250 (Magoc and Salzberg, 2011). The merged reads were then subjected to dereplication and formatting using the UPARSE workflow (Edgar, 2013). Dereplicated reads were clustered using the default settings of the ‘usearch v. 7.0.1090-cluster_otus’ command. Operational Taxonomic Unit (OTU) abundances were estimated using the ‘usearch v. 7.0.1090-search global’ command with-id 0.97-maxacepts – -maxrejects 0. Taxonomic assignment was performed using the uclust classifier, implemented in the assign_taxonomy.py script in QIIME (Caporaso et al., 2010), and the MiDAS database, release 481 (McIlroy et al., 2017). Bioinformatic analyses were conducted in RStudio IDE (version 1.4.1717), utilizing R packages such as ampvis (version 2.7.8), tidyverse (version 1.3.1), seqinr (version 4.2.8), ShortRead (version 1.50.0), and iNEXT (version 2.0.20) (Chao et al., 2014; Hsieh et al., 2016).

## 3 Results

### 3.1 ALE variation in different types of sludge

**Figure 1:**
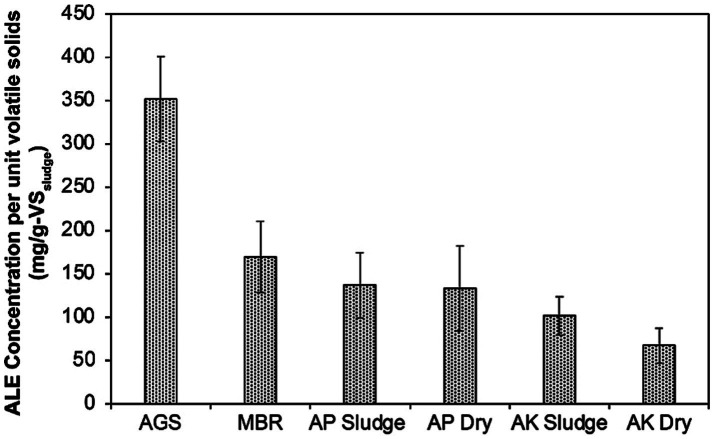
Comparing the ALE concentrations in mg/g-VS across sludge types from different wastewater treatment processes. AGS is referred to as AGS sludge, MBR as MBR sludge, AP denotes the CAS plant treating domestic wastewater, labeled as AP Sludge (undried) and AP Dry (dried). AK denotes the CAS plant treating combined industrial and domestic wastewater, labeled as AK Sludge (undried) and AK Dry (dried).

Upon visual inspection, the extracted ALE from AGS granules and MBR sludge showed a noticeable color difference, with the AGS ALE appearing lighter than the dark brown MBR ALE (Supplementary Figure S1). This visual difference is probably caused by varying ALE content in the two types of sludge, suggesting diverse potentials for resource recovery and applications. In quantitative terms, the AGS sludge exhibited a higher concentration of ALE per unit volatile solids (352 mg/g-VS_sludge_) compared to the MBR sludge (170 mg/g-VS_sludge_), as illustrated in Figure 1.

### 3.2 Impact of wastewater treatment process on the composition of ALE

Previous studies have demonstrated the ability of ALE extracts to form hydrogels (Schambeck et al., 2020a; Lin et al., 2010). To obtain soluble sodium alginate (Na^+^-ALE) 0.1 M NaOH was gradually added to the precipitated ALE until the pH 8.5 is reached. The solubilized Na^+^-ALE was then dripped into a 2.5% CaCl_2_ solution using a Pasteur pipette. Upon contact with the CaCl_2_ solution, the Na^+^-ALE formed circular beads as illustrated in Supplementary Figure S2, demonstrating its ability to form an ionic hydrogel (Schambeck et al., 2020a).

**Figure 2:**
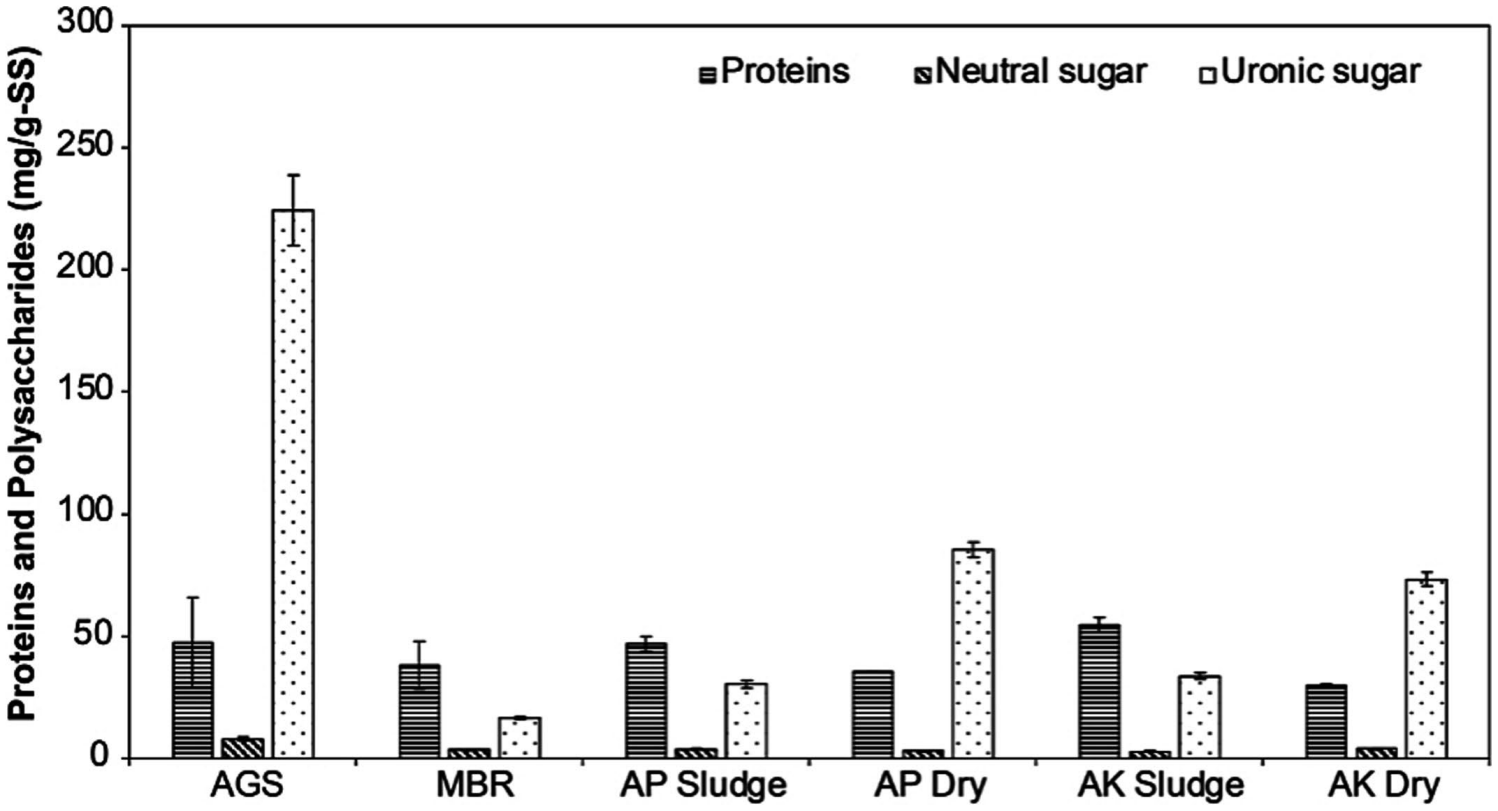
Quantification of protein, neutral sugar, and uronic sugar concentrations in mg/g-SS across six different sludge types. AGS is referred to as AGS sludge, MBR as MBR sludge, AP denotes the CAS plant treating domestic wastewater, labeled as AP Sludge (undried) and AP Dry (dried). AK denotes the CAS plant treating combined industrial and domestic wastewater, labeled as AK Sludge (undried) and AK Dry (dried). AGS-derived ALE shows the highest uronic acid content, which is crucial for hydrogel stability, while MBR-derived ALE has a higher protein content.

As previously reported, our results also show that Na^+^-ALE contains polysaccharides and proteins (Figure 2). The composition of Na^+^-ALE was found to vary depending on the sample source. Protein concentrations were between 30 and 54 mg/g of VS sludge. Polysaccharide concentrations, quantified in terms of uronic acids, varied significantly among samples. The lowest concentration of uronic acids was in MBR sludge (16.5 ± 0.4 mg/g of SS sludge), while the highest concentration was observed in Na^+^-ALE from AGS (224 ± 14.5 mg/g of VS sludge). Neutral sugars were detected in minimal amounts, ranging from 3 to 8 mg/g of VS sludge, as depicted in Figure 2.

In contrast to recent findings by Schambeck et al. (2020a), which suggest proteins are the most abundant component of ALE, this study reveals that the relative abundance of proteins and polysaccharides varies with the source of the wastewater. Polysaccharides were found to be the predominant component of ALE extracted from dry sludge from municipal and industrial wastewater treatment plants, exhibiting concentrations approximately 2.5-fold higher than those of proteins. In AGS, the concentration of polysaccharides was even more pronounced, being 5-fold higher than that of proteins, confirming the findings of Lin et al. (2010). Conversely, wet sludge from MBR and conventional activated sludge treatment plants, which process municipal and industrial wastewater, exhibited a 1.5 to 2-fold higher protein concentration compared to polysaccharides. Furthermore, the results confirm that uronic acids were present in higher concentrations than neutral sugars in all samples, aligning with the findings of Schambeck et al. (2020a). Notably, AGS showed the highest concentration of uronic acids, being at least 2.6 times greater than in other samples (Figure 2).

### 3.3 Glycosyl composition and molecular weight analysis

Glycosyl composition analysis using GC–MS indicates that ALE from different WW is primarily composed of rhamnose, mannose, galactose, glucose, and N-acetyl-glucosamine (Table 1). Other monosaccharides such as ribose, arabinose, fucose, xylose, galacturonic acid, and N-acetyl-mannosamine were detected in relatively small amounts. The uronic acids, glucuronic acid, and galacturonic acid were detected in small amounts compared to neutral sugars. These results appear to contradict our chemical analysis showing a higher content of uronic acids in ALE samples than in neutral sugars (Figure 2). The high abundance of uronic acids detected using chemical methods may be the result of the combined effect of different uronic acids and the higher content of mannuronic acid. However, we were unable to quantify mannuronic acid in the GC–MS data due to a lack of standards. ALE from AGS and AP Dry was rich in glucose (48 and 21% respectively), whereas ALE from MBR showed a higher content of mannose (32%) followed by glucose (16%). The ALE from AK Sludge, AK Dry, and AP Sludge was dominated by rhamnose (20, 20, and 17% respectively), followed by glucose. Rhamnose was detected in EPS produced by floc-forming bacteria commonly detected in WW (Allen et al., 2004). As expected, these results show that WW composition in terms of nutrients and microbial community has a strong effect on the monomeric composition of polysaccharides. The variations in monosaccharide composition further suggest that ALE derived from different sludge sources may exhibit distinct structural and functional properties, influencing their application potential. For instance, the higher glucose content in AGS-derived ALE may enhance its gelling properties, while the higher mannose content in MBR-derived ALE could affect its viscosity and binding capacities.

**Table 1:** Glycosyl composition (mole %) and carbohydrate percentage of EPS samples.

Glycosyl residue	AK dry (mole%)	AK sludge (mole%)	AP dry (mole%)	AP sludge (mole%)	MBR (mole%)	AGS (mole%)
Ribose (Rib)	2.3	2.0	1.7	2.8	1.5	0.2
Arabinose (Ara)	6.8	3.2	6.9	9.6	7.9	0.9
Rhamnose (Rha)	20	19.6	16.3	16.6	9.9	12.4
Fucose (Fuc)	4.2	3.7	3.8	4.1	2	7.1
Xylose (Xyl)	7.5	2.9	3.3	7.0	5.6	0.5
Glucuronic Acid (GlcA)	2.1	0.2	2.5	0.4	0.3	1.0
Galacturonic Acid (GalA)	4.0	2.0	3.3	3.7	6.0	2.0
Mannose (Man)	17.3	11.5	11.2	10.5	31.9	3.8
Galactose (Gal)	13.8	10.7	11.6	12.5	5.4	4.6
Glucose (Glc)	17.1	18.5	21.2	6.6	15.6	48.4
Heptose (Hep)	1.2	1.3	0.8	0.05	1.0	1.2
N-acetylgalactosamine (GalNAc)	n.d.	1.4	1.5	1.9	1.4	n.d.
N-acetylglucosamine (GlcNAc)	2.5	22.1	13.8	18.7	9.1	15.9
N-acetylmannosamine (ManNAc)	1.2	0.9	2.1	5.6	2.3	2.1
Total	100	100	100	100	100	100
%CHO	5.1	3.4	5.9	6.0	7.0	5.0

%CHO, carbohydrate percentage; n.d., not detected. AGS is referred to as AGS sludge, MBR as MBR sludge, AP denotes the CAS plant treating domestic wastewater, labeled as AP Sludge (undried) and AP Dry (dried). AK denotes the CAS plant treating combined industrial and domestic wastewater, labeled as AK Sludge (undried) and AK Dry (dried).

The ALE was subjected to Size-Exclusion Chromatography (SEC), which showed a similar molecular weight distribution of polysaccharides. All the samples consist of high molecular weight polysaccharides ranging in size from more than 700 KDa to 500 KDa (Supplementary Figures S3–S9). The ALE from AK Dry has a predominant peak in the high molecular weight range compared to other samples. In addition, samples also consist of lower molecular weight species ranging from 40 KDa to less than 1 KDa. It is important to note that the samples were filtered using 0.4 μm nylon filters which may have resulted in some polysaccharides being filtered out. Therefore, it should be considered that the estimated weights may not represent the full sample. The molecular weight of ALE polysaccharides was higher than the molecular weight of polysaccharides produced by biofilm-forming seawater bacteria (Rehman et al., 2021). It was suggested that the high molecular weight polysaccharides may serve as a scaffold for the attachment of proteins, lipids, and nucleic acids together forming the biofilm matrix (Bales et al., 2013). Comparing the ALE polysaccharides with a commonly studied exopolysaccharide, seaweed-derived alginates, highlights their distinct properties. Seaweed alginates are primarily composed of mannuronic and guluronic acids, which contribute to their strong gelling properties (Abka-Khajouei et al., 2022). In contrast, the higher content of neutral sugars in ALE suggests different gelling and binding behaviors, offering potential for unique applications in industrial processes that require alternative functional characteristics.

### 3.4 Nuclear magnetic resonance spectroscopy

**Figure 3:**
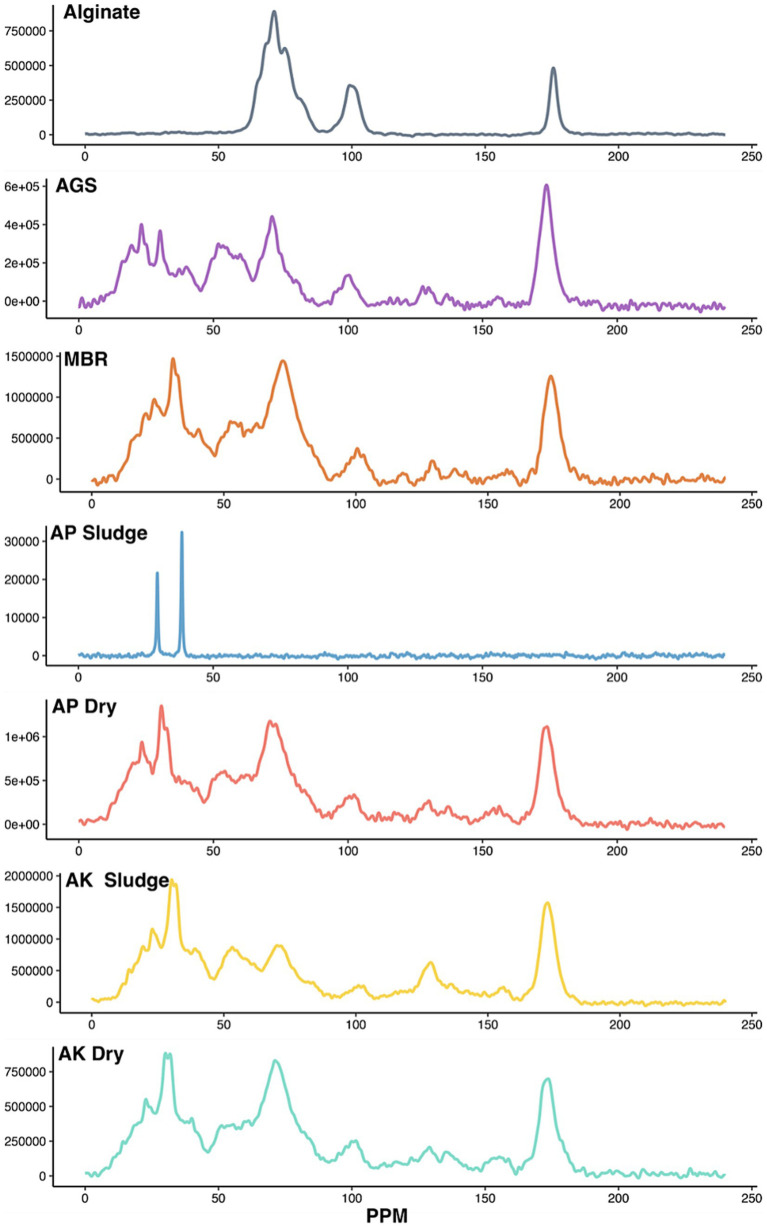
NMR analysis of the ALE as compared to pure alginate. Solid-state ^13^C NMR spectra reveal chemical structures in the ALE extracted from various sludge samples. *Y*-axis represents signal intensity in arbitrary units. AGS is referred to as AGS sludge, MBR as MBR sludge, AP denotes the CAS plant treating domestic wastewater, labeled as AP Sludge (undried) and AP Dry (dried). AK denotes the CAS plant treating combined industrial and domestic wastewater, labeled as AK Sludge (undried) and AK Dry (dried).

We performed solid-state ^13^C NMR spectroscopy to identify the different functional groups associated with EPS (Figure 3). The electronic structure of the immediate environment of ^13^C carbon atoms determines their resonance frequencies. For example, NMR analysis shows that aliphatic carbons resonate in the frequency range of 0–40 ppm, secondary alcohols of carbohydrates resonate in the frequency range of 60–90 ppm, and carbon atoms of glycoside bonds resonate in the frequency range of 95–106 ppm (Jiao et al., 2010). Moreover, the resonance frequency also indicates glycosidic linkage, i.e., alpha or beta oxygen linkages. Carbon atoms forming linkages to alpha oxygen resonate in the range of 95–103 ppm, while linkages to beta oxygen resonate in the range 103–106 ppm (Rehman et al., 2021; Jiao et al., 2010).

The peak observed at 20 ppm suggests methyl carbon (Khan et al., 2014), while the peak at 30 ppm indicates methylene groups in the long-chain hydrocarbons (Jiao et al., 2010; Metzger et al., 2009), suggesting the presence of hydrophobic compounds in EPS. These hydrophobic compounds in EPS usually originate from phospholipids and lipopolysaccharides of the bacterial cell membrane. These peaks were absent in pure alginate (top) further suggesting that the hydrophobic compounds in EPS originate from microbial structures. Similar to previous findings [39], the EPS from AP Dry, AK Dry, AK Sludge, AGS, and MBR contains lipids, albeit the proportion of lipids for each ALE remains to be determined (Figure 3). Furthermore, the 30 ppm peak was less pronounced in AGS.

### 3.5 Microbial community analysis

**Figure 4:**
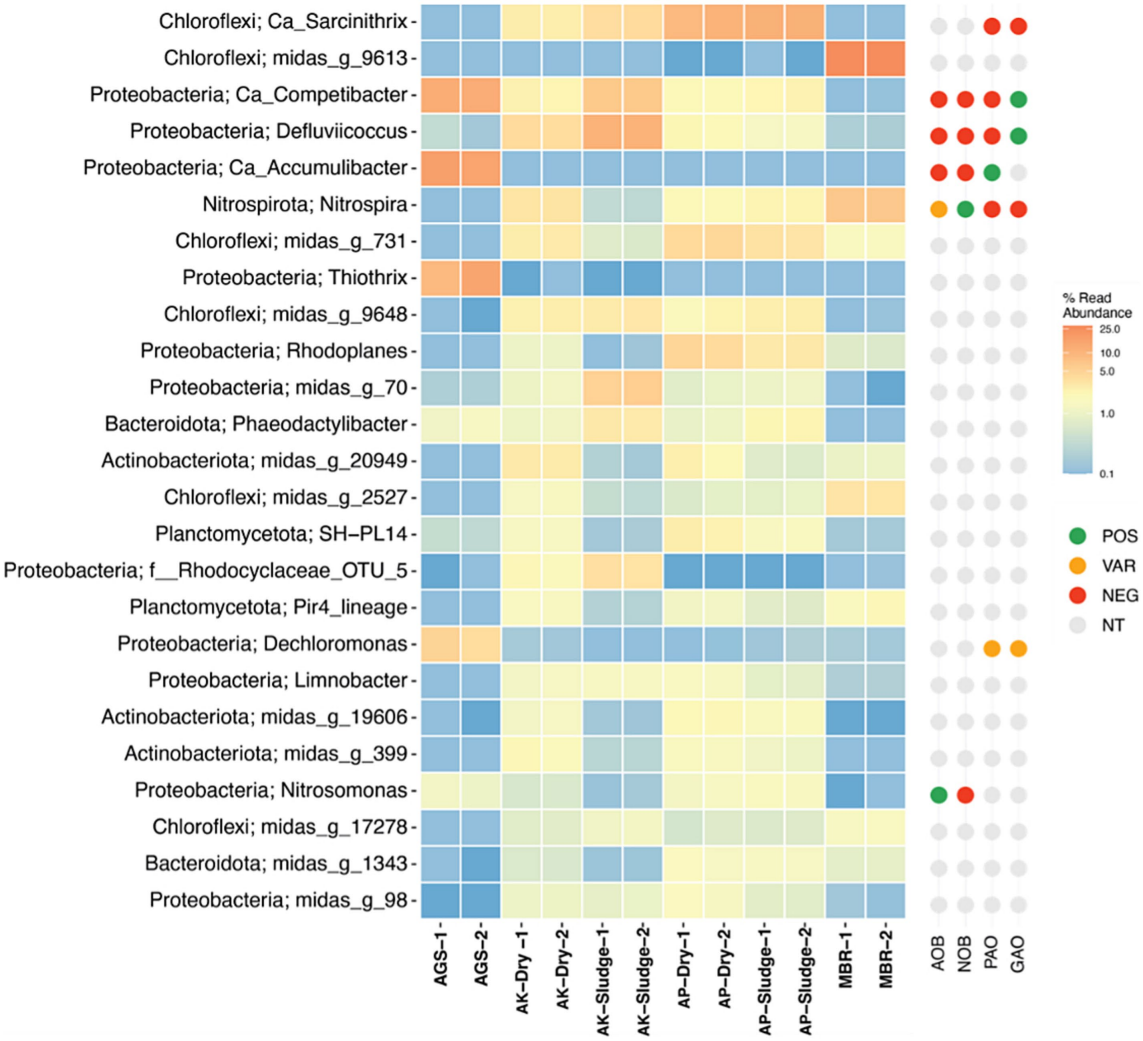
Heatmap distribution of the most dominant OTUs representing ≥1.0% abundance, classified to the lowest taxonomic level possible (g: genus, f: family, o: order, c: class, p: phylum) across various waste sludges. The right panel next to the heatmap displays key functional group information, sourced directly from midasfieldguide.org. These groups include [AOB (Ammonia-oxidizing bacteria), NOB (Nitrite-oxidizing bacteria), PAO (Polyphosphate-accumulating organism), and GAO (Glycogen-accumulating organism)]. AGS is referred to as AGS sludge, MBR as MBR sludge, AP denotes the CAS plant treating domestic wastewater, labeled as AP Sludge (undried) and AP Dry (dried). AK denotes the CAS plant treating combined industrial and domestic wastewater, labeled as AK Sludge (undried) and AK Dry (dried). Detailed species-level OTU is presented as Supplementary Table S1.

The composition of microbial communities in WWTP is known to vary based on factors, including wastewater composition, process configuration, and environmental conditions (Xie et al., 2021). It was observed that the microbial communities in AGS and MBR were significantly different from those in CAS-based WWTPs (Figure 4). Similarly, the microbial community composition of the AGS system was significantly different from the MBR system. Interestingly, there were no significant differences in community compositions in CAS plants treating either municipal or industrial wastewater. These results suggest that the microbial community composition is more influenced by the treatment process than by the source of the wastewater.

Previous studies have recognized Proteobacteria as the most dominant phyla in WWTPs (Rehman et al., 2019; Fortunato et al., 2018; Rehman et al., 2020). Similarly, this study found Proteobacteria to be the most abundant in AGS and AK plants treating industrial wastewater, as illustrated in Figure 4. *Proteobacteria* play a key role in WWTP microbial communities, aiding in the degradation of organic pollutants and denitrification (Xie et al., 2021). Among the *Proteobacteria*, *Pseudomonas alcaligenes* was found to be particularly abundant in the AGS system. This species is known for its potential to synthesize alginate, and its presence may be closely associated with the higher production of ALE in AGS-derived sludge. The ability of *Pseudomonas alcaligenes* to form biofilms and its role in polysaccharide production could enhance the recovery of ALE, highlighting the species’ significance in this context. *Chloroflexi* were predominantly observed in AP and MBR plants processing domestic wastewater. In a previous lab-scale bioreactor study, *Chloroflexi* emerged as the second most abundant bacterial phylum in MBR sludge (Miura et al., 2007). The variations in microbial communities between lab-scale setups and full-scale plants may be attributed to differences in process parameters and environmental conditions. These findings confirm *Chloroflexi*’s prominence in wastewater microbial communities. Notably, *Chloroflexi* sp. *midas_g_9613* was prevalent in MBR samples, while *Chloroflexi* sp. and *Ca_Sarcinithrix* dominated in AP sludge. *Chloroflexi*’s importance in breaking down polymers and complex organic substances into simpler molecules for other microbes was emphasized by Zhang et al. (2017). The filamentous nature of *Chloroflexi*, which contributed to its higher abundance in MBR due to its ability to penetrate between the membrane and foulants, was noted by Jo et al. (2016). Additionally, Phosphate-accumulating and Glycogen-accumulating *Ca_Accumulibacter* were mainly found in AGS sludge, indicating AGS’s effectiveness for phosphate removal. A diverse array of *Ca_Accumulibacter* was predominantly detected in AGS samples.

## 4 Discussion

Alginate is a valuable polysaccharide produced on a commercial scale using seaweed. It has found applications in the food, cosmetic, pharmaceutical, and medical industry. In the food industry, alginate is commonly used as a thickening, gelling, stabilizing, or emulsifying agent. Recently, alginate use has expanded to pharmaceutical and biomedical industries due to its biocompatibility, biodegradability, cell affinity, and wound healing properties (Gheorghita Puscaselu et al., 2020). Alginate production is primarily attributed to two bacterial genera, *Pseudomonas* and *Azotobacter* (Hay et al., 2013; Hay et al., 2014; Maleki et al., 2016; Rehman and Rehm, 2013). This study investigated the prevalence of these genera in sludge samples from various wastewater treatment plants, with a hypothesis suggesting that AGS would have a higher abundance of either genus due to its elevated ALE content. The analysis revealed that *Azotobacter* was not detected in any samples, while *Pseudomonas* and its five species were present (Supplementary Table S1). Among these, four species were identified in the MiDAS taxonomy database (Dueholm et al., 2022). The fifth species, *Pseudomonas alcaligenes*, known for its genetic potential to synthesize alginate and form biofilm (Pintado et al., 2021), was most abundant in AGS sludge. In contrast, only one measure of *P. alcaligenes* was found in a sample from the activated sludge plant treating industrial sewage, suggesting a potential correlation between the prevalence of *P. alcaligenes* and the higher ALE content in the AGS process. This finding highlights the importance of further investigating the role of *P. alcaligenes* in ALE production and its potential significance in AGS-based wastewater treatment.

By elucidating the prevalence of *P. alcaligenes* in AGS sludge and its association with ALE production, this study enhances the current understanding of alginate synthesis in wastewater treatment processes. The identification of *P. alcaligenes* as a potential key producer of ALE opens up new possibilities for the targeted manipulation of microbial communities to enhance ALE yields. Moreover, this approach may result in more efficient resource recovery from sludge. Given the higher ALE yield and uronic acid content in AGS sludge, the resulting ALE could be particularly valuable in industries requiring strong gelling properties, such as the pharmaceutical and biomedical sectors. In contrast, ALE from MBR sludge, with its lower uronic acid content but higher flexibility due to its polysaccharide profile, may be more suited for applications in the food and cosmetic industries, where flexibility and emulsification are important.

The uronic acid and protein levels observed in this study are comparable to those reported in other systems. The uronic acid content in ALE derived from AGS, at 224 mg/g-VS, corresponds with values reported by Lin et al. (2013) for aerobic granular sludge systems. Schambeck et al. (2020a) also reported similar uronic acid levels in ALE from activated sludge systems, though they highlighted differences in composition between AGS and CAS systems. However, the uronic acid content in ALE from MBR sludge in this study was lower than previously reported for flocculant sludge, likely reflecting differences in microbial communities and wastewater composition across treatment processes. Protein levels measured in this study (30–54 mg/g-VS) align with those observed in EPS from both activated sludge and biofilm systems. Chen et al. (2022) reported similar protein concentrations in EPS extractions from bacterial and algal-bacterial AGS. The elevated protein levels in MBR-derived ALE (up to 54 mg/g-VS) are consistent with findings by Liu et al. (2023), who noted improved recovery of ALE using sodium percarbonate, suggesting that variations in extraction methods can influence the recovery and composition of biopolymers like proteins. These variations highlight the influence of treatment processes on the polysaccharide and protein composition of ALE.

The environmental benefits of ALE recovery from WWTPs are also notable. By extracting ALE from waste sludge, WWTPs could reduce the overall sludge volume, which would lower disposal and transportation costs and mitigate the environmental impacts of sludge management. This process also enhances resource recovery by converting waste into valuable biopolymers that have diverse applications in industry. These factors contribute to the sustainability of WWTPs and support the development of circular economy models.

Additionally, these findings emphasize the importance of considering specific microbial species and their functional attributes in the design and optimization of wastewater treatment systems. This is in contrast to prior studies, which mainly focused on characterizing alginate-producing bacteria in controlled laboratory environments and specific settings (Meena et al., 2020). While these studies provide valuable insights into the genetic and physical potential of various bacterial species to produce alginate, they often overlook the complex ecological interactions and synergies among microbial species within active WWTPs. These interactions are crucial, as they significantly affect the properties and yield of ALE recovered. Building upon this knowledge, the current study contributes to the development of more sustainable and efficient wastewater treatment processes. The insights obtained here can inform the design of innovative treatment strategies that exploit the potential of ALEs for resource recovery, thus facilitating the advancement of more eco-friendly wastewater management practices.

Further research should focus on the mechanisms by which *P. alcaligenes* influences ALE production in AGS sludge. A more comprehensive understanding of the genetic regulation and environmental factors that enhance alginate synthesis in this bacterial species could lead to novel strategies for increasing overall ALE yields in WWTPs. Additionally, exploring the potential of *P. alcaligenes* to form biofilms may shed light on the role of biofilm dynamics in ALE production. Acquiring such knowledge is crucial for integrating biofilm management strategies into effective sludge management approaches.

The NMR spectra confirmed the presence of secondary alcohols and revealed the type of glycoside bonds linking the monomers. The peak at 72 ppm, indicative of secondary alcohol carbon, was detected in all the ALE samples (Khan et al., 2013). This is the most dominant peak in alginate samples, which is a purified polysaccharide. The peaks at 90 ppm and 102 ppm indicated that carbohydrates in all the samples, except pure alginate, contain *α*-glycoside bonds (Figure 3). The alginate consists of beta-D-mannuronic acid and alpha-L-guluronic acids linked by beta1-4, glycosidic bonds (Remminghorst and Rehm, 2006). The ALE samples consist of a mixture of polysaccharides produced by diverse bacteria likely containing polysaccharides containing alpha and beta-glycosidic bonds. However, the peaks at 90 ppm and 102 ppm are of varying intensity in all the samples. The peaks at 130 ppm and 158 ppm were indicative of proteins containing hydroxy aromatic amino acids, such as tyrosine (Khan et al., 2014; Khan et al., 2013). These peaks were detected in all the ALE samples except for pure alginate as expected. A sharp peak at 173 ppm (Figure 3) in all samples except alginate indicated the presence of an amide carbon, further confirming the presence of proteins in the sample (Jiao et al., 2010). Finally, all the samples share peaks at 72 ppm and 100 ppm with alginate, indicating all ALE samples contain alginate-like polysaccharides.

Quantities and chemical and physical properties of ALE are influenced by the wastewater treatment process, sludge type, and wastewater composition. Our study shows that the AGS process yields higher quantities of ALE with superior chemical and physical properties, attributed to the unique microbial consortium in the system. These findings are valuable for resource recovery from wastewater and for identifying suitable industrial applications for ALE extracted from various wastewater treatment processes.

These findings advance the field of resource recovery by providing a deeper understanding of how different wastewater treatment processes, particularly AGS systems, influence ALE production. The high ALE yield and superior properties associated with AGS demonstrate its potential for resource recovery applications. This knowledge can inform future technologies by highlighting the importance of microbial community optimization, specifically targeting species such as *Pseudomonas alcaligenes* to enhance ALE production. Future research could explore genetic modifications in microbial communities, such as enhancing the alginate synthesis pathways in *Pseudomonas alcaligenes*, to further increase ALE production. Additionally, ALE extraction techniques could be applied to other types of waste, including agricultural residues or industrial by-products, to evaluate their potential for ALE recovery. Further studies could also focus on optimizing the extraction processes by testing new solvents or adjusting operational conditions to improve ALE yield. Finally, scaling up ALE production in full-scale wastewater treatment plants would be essential for assessing its economic feasibility and environmental impact on a larger scale. By focusing on these microbial and process-related factors, future wastewater treatment systems could be designed not only to improve resource recovery but also to promote more sustainable and efficient circular economy models. These insights pave the way for innovative wastewater management technologies that can reduce costs, improve sustainability, and expand the industrial use of ALE.

## Data Availability

The raw sequencing data generated in this study are deposited at the National Center for Biotechnology Information (NCBI) database, accessible to the public under the accession number PRJNA860785.
