# Supplementary material for: Alginate-like exopolysaccharides extracted from different waste sludges exhibit varying physicochemical and material properties
Source: Front Microbiol. 2024 Nov 5;15:1493782. doi: 10.3389/fmicb.2024.1493782 (PMC11573756; doi:10.3389/fmicb.2024.1493782)
Supplement: Supplementary file 1 [file Data_Sheet_1.docx]

Supplementary Material

# Supplementary Figures and Tables

## Supplementary Figures


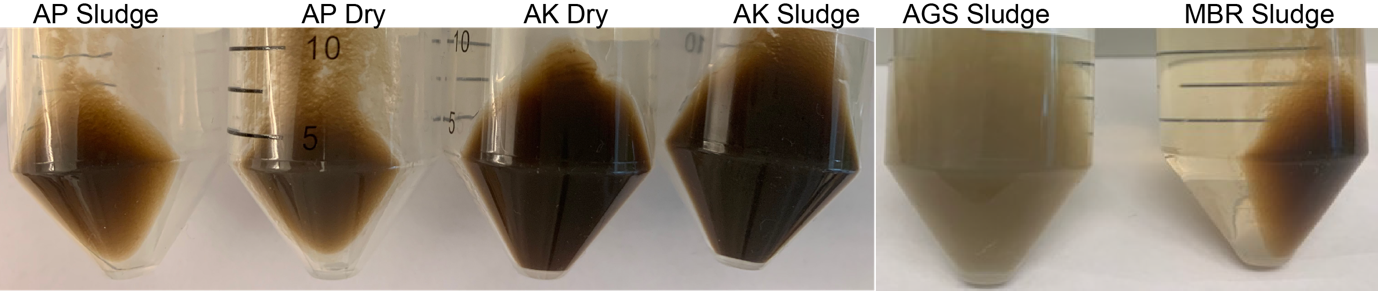


**Figure S1.** Visual comparison of sludge samples obtained from different wastewater treatment plants. AGS is referred to as AGS sludge, MBR as MBR sludge, AP denotes the CAS plant treating domestic wastewater, labelled as AP Sludge (undried) and AP Dry (dried). AK denotes the CAS plant treating combined industrial and domestic wastewater, labelled as AK Sludge (undried) and AK Dry (dried).


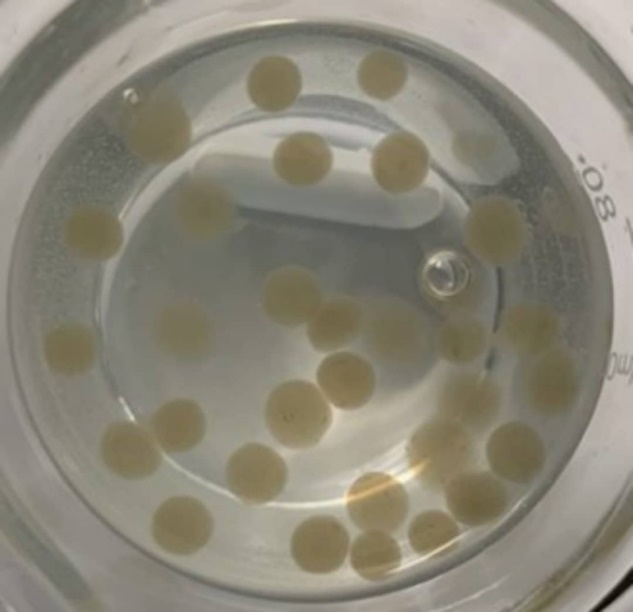


**Figure S2.** Formation of large circular Na^+^-ALE beads upon being in contact with a 2.5% CaCl_2_ solution.

**Figure S3.** HPLC chromatogram of dextran standards used for molecular weight determination of EPS samples by SEC analysis (1740 kDa [void volume - Vo], 500 kDa, 167 kDa, 40 kDa, 10 kDa, and 1 kDa).

**Figure S4.** HPLC chromatogram of sample AK-D obtained by SEC.

**Figure S5.** HPLC chromatogram of sample AK-S obtained by SEC.

**Figure S6.** HPLC chromatogram of sample AP-D obtained by SEC.

**Figure S7.** HPLC chromatogram of sample AP-S obtained by SEC.

**Figure S8.** HPLC chromatogram of sample MBR obtained by SEC.

**Figure S9.** HPLC chromatogram of sample AGS obtained by SEC.
